# Follicular size predicts success in artificial insemination with frozen-thawed sperm in donkeys

**DOI:** 10.1371/journal.pone.0175637

**Published:** 2017-05-17

**Authors:** Joseph Saragusty, Alemayehu Lemma, Thomas Bernd Hildebrandt, Frank Göritz

**Affiliations:** 1 Department of Reproduction Management, Leibniz Institute for Zoo and Wildlife Research, Berlin, Germany; 2 College of Veterinary Medicine and Agriculture, Addis Ababa University, Debre Zeit, Ethiopia; Universite Blaise Pascal, FRANCE

## Abstract

In asses, semen collection, cryopreservation, and artificial insemination (AI) with frozen-thawed semen have been scarcely described and success rate, particularly following AI, is reportedly low. In the absence of reliable protocols, assisted reproductive technologies cannot support the conservation efforts aimed at endangered wild ass species and domestic donkey breeds. Two experiments were conducted in this study. In experiment 1 we evaluated freezing Abyssinian donkey (*N* = 5, 4 ejaculates each) spermatozoa using three freezing extenders (Berliner Cryomedium + glycerol, BC+G; BotuCrio, BOTU; INRAFreeze, INRA) and two cryopreservation techniques (liquid nitrogen vapour, LNV; directional freezing, DF). Post-thaw evaluation indicated that BOTU and INRA were similar and both superior to BC+G (*P* ≤ 0.004 for all motility tests), and that DF was superior to LNV (P < 0.002 for all evaluation parameters). In experiment 2, relying on these results, we used Abyssinian donkey sperm frozen in BOTU and INRA by DF for AI (*N* = 20). Prior to AI, thawed samples were diluted in corresponding centrifugation media or autologous seminal fluids at 1:1 ratio. No difference was found between BOTU and INRA or between the addition of seminal fluids or media, all resulting in ~50% pregnancy, and no differences were noted between males (*N* = 4). The size of pre-ovulatory follicle was a significant (*P* = 0.001) predictor for AI success with 9/10 pregnancies occurring when follicular size ranged between 33.1–37.4 mm, no pregnancy when it was smaller, and only one when larger. A number of ass species face the risk of extinction. Knowledge gained in this study on the Abyssinian donkey can be customised and transferred to its closely related endangered species and breeds.

## Introduction

The genus *Equus* includes four species of donkeys—the African wild ass (*Equus africanus*; critically endangered), the Asiatic wild ass (also known as the Onager; *E*. *hemionus*; endangered), the Kiang (*E*. *kiang*; least concern), and the domestic donkey (*E*. *asinus*) with 128/162 breeds (79%) under the threat of extinction [1–3]. Yet, very little is known about donkeys’ reproduction. In many cases the populations are so small, fragmented and dispersed, that the only way to prevent excessive inbreeding with its dire consequences is to resort to the aid of assisted reproductive technologies [4]. Cryopreservation of germplasm (gametes and embryos) of threatened or endangered species has been proposed as an approach to slow or halt the rate of species decline [5,6]. Artificial insemination (AI) with frozen-thawed sperm would allow dissemination of genetic material among members of each species and breed across studs, farms, zoos, and sanctuaries around the world, as well as between the captive and wild populations, as we have previously demonstrated in African elephants [7] and the European brown hare [8].

The first equine foal conceived through AI with frozen-thawed sperm was born in 1957 [9]. The situation is lagging far behind in the closely related domestic donkey and its wild relatives. While much has been done and written on cryopreservation of stallion semen, only a handful of studies could be found in the scientific literature on issues related to donkey semen cryopreservation. These includes reports on several donkey breeds such as the Baudet Du Poitou [10–14], Zamorano-Leonés [15,16], Pêga [17–19], Martina Franca [20,21], Catalonian [13,22], Grand Noir du Berry [13], Andalusian [23–25], Amiata [26,27], Abyssinian (Lemma et al., unpublished data), the American standard breed [28], and recently also for the onager [29,30]. Post-thaw sperm quality in these studies, as judged by laboratory evaluation techniques, was fair to excellent. However, in the few studies in which AI was attempted, success rate was very limited and the target females were mostly mares rather than jennies [13]. Glycerol was suggested to be a major factor contributing to failures in both the donkey and the horse [11,13]. In stallions and jacks it was also shown that adding seminal fluids could improve the fertility of their frozen-thawed semen [26,31]. From these studies, however, no clear and consistent procedure has emerged and further studies are clearly needed to make AI and sperm banking standard procedures in the efforts to preserve the different endangered donkey species, subspecies and breeds.

One assisted reproductive technology that has never been tested for donkey sperm cryopreservation is the multi-thermal gradient directional freezing (DF) technology. This technology was shown in several studies, conducted by us and others, to consistently produce good results in cryopreservation of spermatozoa from a wide variety of species including domestic species such as cattle [32,33], horses [34] and goat buck [35], and wildlife species such as elephant [36,37], gazelle [38], hippopotamus [39], rhinoceros [40], whale [41], dolphin [42] and many others (see [43] for a recent review). A number of studies have demonstrated that the multi-thermal gradient directional freezing technology is superior to conventional freezing techniques (e.g. [29,34,42,44]). In relevance to this study on asses, we showed in stallions that while 88% of the ejaculates (85/97) showed over 35% post-thaw progressive motility when frozen by the directional freezing technique, only 59% (57/97) of the ejaculates showed such acceptable motility when frozen by the conventional liquid nitrogen vapour technique [34]. In that study, the directional freezing technique was also shown to be superior to the liquid nitrogen vapour freezing technique in average post-thaw motility (50.2% vs. 37.4%), viability (53.6% vs. 39.5%) and membrane integrity as evaluated by the hypo-osmotic swelling test (36.2% vs. 26.5%). These studies clearly demonstrate the high potential the directional freezing has for cryopreservation of donkey sperm.

Based on the available literature and many years of experience working with the directional freezing technology, we believe that using this technology with some modifications to the current freezing media and AI procedure, success rate in artificial insemination of jennies with frozen-thawed jacks’ sperm can be improved. The experiments described here were designed with this objective in mind.

## Materials and methods

### Ethical considerations

The College of Veterinary Medicine and Agriculture (CVMA) of Addis Ababa University, Ethiopia, as a teaching, research, and community service rendering institution, has the mandate to house, breed, and experiment with animals. All activities involving animals (semen collection, artificial insemination, pregnancy evaluation) were performed by and under the strict supervision of veterinarians with long-standing experience, following approval of the project by the CVMA. These activities are non-invasive procedures performed routinely in domestic and often also in non-domestic species, are integrated into husbandry and breeding guidelines, and require no specific ethical permit. Privately owned jennies participated in the study only if their owners gave their full consent after being briefed in detail about the study, the specific procedures to be performed on their animals and the potential risks and benefits.

### Animals

A total of 25 domestic Abyssinian donkeys participated in this study.

Five jacks, aged between 7 and 10 years and with at least one foaling experience based on information provided by their seller, acted as semen donors. All jacks were housed at the stable of the CVMA. Twenty parous jennies, aged 3.5 to 20 years, were recruited for the study. Of these, three were owned by the CVMA and the rest privately owned by local farmers. The breeding history, reproductive soundness, general health and body condition of each jenny were taken into consideration during recruitment into this study. Age of the selected animals, when not known to the owner, was determined by dentition [45]. All animals participating in this study were of body condition 4.0–5.0 according to the established Pearson and Ouassat scoring system [46]. Animals at the CVMA were free to graze during the day and their diet was supplemented by hay and wheat bran. They had access to water *ad libitum*. Privately owned animals were kept at their owners’ home, without any contact with males for the duration of the study. Animals had free access to water, were free to graze and their diet supplemented with straw and cereal by-products. All animals were in good body condition throughout the study.

### Semen collection

Semen was collected by letting the jacks mount on an oestrous female and using Colorado model equine artificial vagina (Agtech, Inc., Manhattan, KS, USA) with disposable liner (Minitube, Tiefenbach, Germany) connected to a collection bottle lined with sterile Whirl-Pak^®^ sampling bag (Minitube) and disposable filter (Minitube). Each jack was collected five times with 3–4 days rest between consecutive collections. The jacks had no previous training for the procedure. All jacks responded well and semen was normally collected within 20–30 min.

### Semen evaluation

Samples were evaluated for total and progressive motility, vigour, volume, concentration, viability, and morphology. Evaluations were performed after collection, before centrifugation, and again after thawing. Motility was also assessed 3 h after thawing and incubation at 37°C. Total ejaculate volume was assessed using a graduated tube. Sperm concentration was estimated using Neubauer haemocytometer. Total and progressive motility were evaluated by phase contrast microscopy at ×100 and × 200 magnifications. A drop of 10 μL of the sample was placed on a pre-warmed (37°C) slide, covered with a pre-warmed cover slip and place on MiniTherm Stage Warmer (Hamilton Thorne, Beverly, MA, USA) set at 37°C on the microscope for evaluation. The same experienced spermatologist evaluated all samples. Vigour was estimated on a scale of 0 to 5 (0 = immotile, 5 = highly active and vigorously motile). Sperm viability was assessed by staining 10 μL of suspended sperm with eosin-nigrosin (eosin Y yellow CI 45380, nigrosin CI 50420 dissolved in 0.9% NaCl; VWR International, Darmstadt, Germany) at a 1:1 ratio, incubating at room temperature for 2 min and then smears were prepared and air-dried before evaluation, which took place within two hours. At least 100 spermatozoa of each sample were evaluated with a light microscope (oil immersion; ×1000). White (unstained) sperm were classified as live and those that showed pink or red coloration were classified as dead. The same slides were also used to assess sperm morphology. Cells were classified as normal, or as having defect in the head, neck, midpiece, or endpiece. Evaluation of sperm morphology included search for a wide range of abnormalities as previously described [39].

### Experimental design

**Fig 1:**
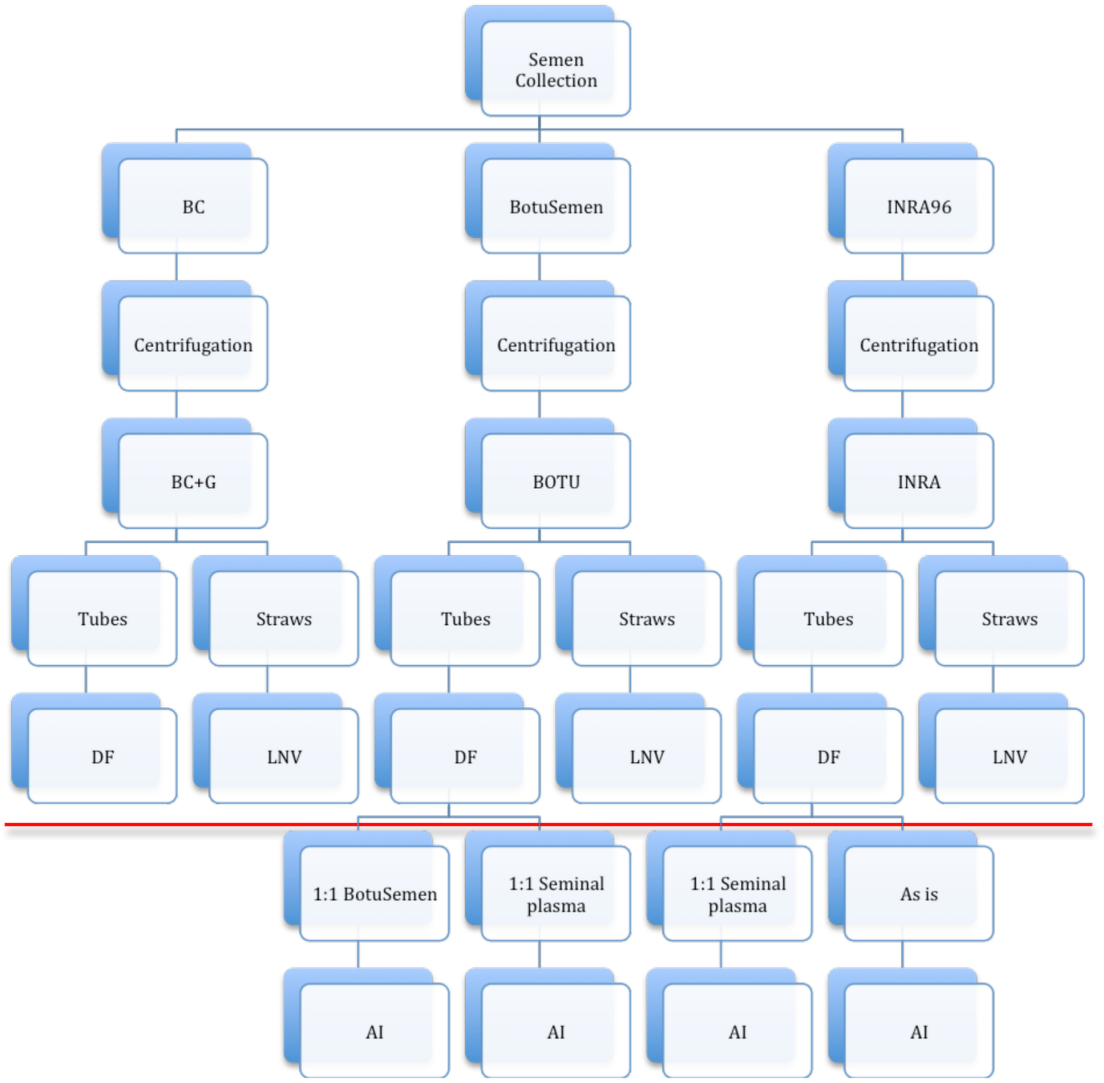
Experimental design flow chart. The study consisted of two stages (experiments). The first stage aimed to assess three freezing extenders (BC+G, BOTU, INRA) and two freezing techniques—directional freezing (DF) and freezing in the vapour phase over liquid nitrogen (LNV). At the second stage, samples frozen with the selected extenders (BOTU and INRA) and freezing technique (DF) were used for AI. After thawing, the BOTU samples were either diluted 1:1 in autologous seminal plasma or in BotuSemen. The INRA samples were either diluted 1:1 in autologous seminal plasma or used as is. A horizontal line on the figure indicates the decision-making point between the two experimental stages.

The study was divided into two experiments that were implemented in a step-wise manner to ensure that the second builds on the previous step’s results (Fig 1).

### Experiment 1: Donkey sperm cryopreservation

This experiment was designed to evaluate the suitability of three freezing extender and two freezing techniques for cryopreservation of jack sperm.

#### Sperm cryopreservation

Of the 20 samples collected, one was deemed unsuitable for freezing (only 10% motility). Good quality samples were processed for freezing as follow: each sample was split into three parts, each diluted 1:1 in one of three different centrifugation media: Berliner Cryomedium (BC) [47], BotuSemen (Nidacon, Mölndal, Sweden), and INRA96 (IMV Technologies, L'Aigle cedex, France). Samples were then underlain with 2 mL of pre-warmed (37°C) 60% iodixanol (OptiPrep^™^; Sigma Aldrich Chemie GmbH, Taufkirchen, Germany), an inert material used as a protective cushion against centrifugation damage [48], and centrifuged at 1000 *g* for 10 min. Following centrifugation, the supernatant and iodixanol were discarded and the pellet was re-suspended in one of three freezing extenders that were matched to the three centrifugation media: BC with glycerol (BC+G, ~4.5% glycerol final concentration), BotuCrio (BOTU; Nidacon), or INRAFreeze (INRA; IMV Technologies) to a final concentration of 200 x 10^6^ spermatozoa/mL. After dilution with the freezing extender, samples were cooled to 4°C over 2 h at ~0.3°C/min by placing the tubes in an isothermal water bath inside a refrigerator. Samples from each extender were split into two parts and packaged into 0.5 mL plastic straws and HollowTubes^™^ (IMT Ltd., Ness Ziona, Israel). Two sizes of HollowTubes^™^ were used: 8 mL for storage as insemination doses, and 2.5 mL for post-thaw evaluation. Straws were frozen by sustaining them at four cm above liquid nitrogen inside a freezing unit’s metal box (Minitube) for 15 min. Straws were then plunged into the liquid nitrogen. HollowTubes^™^ were frozen in a multi-thermal gradient DF machine (MTG-550; IMT Ltd.) as described previously [34,38,49]. All frozen samples were stored under liquid nitrogen pending evaluation and use.

#### Thawing and post-thaw evaluation

Straws were thawed by submerging them in a water bath at 37°C for 30 s. HollowTubes^™^ were thawed by first holding them in the air, at room temperature (24°C) for 90 s and then plunging them into a water bath at 37°C for 60 s. Samples were evaluated immediately after thawing for total and progressive motility, vigour, viability, and morphology, as described above. Remainder of the samples was split into two. One part was kept as is. The other part was diluted 1:1 in the respective centrifugation diluent (BC, INRA96, BotuSemen) to dilute out the cryoprotective agent(s). All samples were incubated at 37°C and evaluated again for motility variables 3h after thawing.

### Experiment 2: Artificial insemination

Based on the results of Experiment 1, only samples frozen by the DF technique with INRA and BOTU as the freezing extender were used in experiment 2. Jennies were randomly split into four equally sized groups for insemination with one of four combinations: i) INRA as is (mean age 11.8±5.8 y); ii) INRA diluted 1:1 with autologous seminal plasma (mean age: 8.8±1.9 y); iii) BOTU diluted 1:1 with BotuSemen (mean age 9.0±3.3 y); iv) BOTU diluted 1:1 with autologous seminal plasma (mean age: 8.3±7.1 y).

#### Seminal plasma collection

Semen was collected once from each of the five jacks for seminal plasma stockpiling. Collected semen was evaluated as described above and then centrifuged without iodixanol at 3000 g for 30 min. The supernatant was alliquoted and stored in insemination portions (8 mL) at -21°C till use.

#### Oestrous induction

Each jenny was evaluated using an ultrasound scanner with a 5MHz linear array transducer (Aloka SSD-500V, Hitachi Aloka Medical, Ltd., Tokyo, Japan) to determine the ovarian status and ascertain they were not pregnant. Jennies were then given a 1.0-mL intramuscular injection of cloprostenol sodium (250 μg/mL Estrumate^®^, Schering-Plough Animal Health Corp, Germany). The jennies were then monitored by ultrasonography for follicular growth and development starting two days after injection and then on a daily basis when the largest follicle has reached the size of 20 mm until ovulation, recording the size of the pre-ovulatory follicle. Endometrial oedema was also monitored as a proxy of estimating approaching ovulation.

#### Artificial insemination

Cryopreserved sperm of four jacks from experiment 1 was used randomly to inseminate 20 jennies. Frozen quantity from the fifth male was insufficient for the multiple inseminations indicated by experimental needs. Only sperm with acceptable motility ≥ 30% was used for the AI procedures. A total of five jennies were assigned to each insemination group as described above. Samples frozen in 8-mL HollowTubes^™^ were thawed, evaluated for motility, and, if suitable, used for insemination. AI was performed only once ovulation was anticipated to occur within the next 24 h during ultrasonographic examination based on pre-ovulatory follicular morphology and endometrial oedema. Each jenny was inseminated twice. Once when the shape and size of the pre-ovulatory follicle, as viewed by ultrasonography, suggested approaching ovulation, and a second after ovulation, which was determined by the disappearance of the preovulatory follicle and the appearance of corpus haemorrhagicum in its place during ultrasonographic examination. In two cases ovulation took an extra day to occur so these jennies were inseminated a third time to make sure all jennies were inseminated twice in proximity to ovulation. Prior to each AI procedure, one insemination dose of 8-mL was thawed, evaluated, prepared with or without the isothermal diluents or seminal fluid, put into a pre-warmed 12 or 24-mL syringe (without plastic plunger), which was then fit onto the insemination catheter (Minitube). The insemination catheter was inserted deep into the uterus where the sperm was deposited. A small amount of air was then pushed through to expulse remaining sperm and to prevent retrograde flux.

#### Pregnancy evaluation

Pregnancy was diagnosed between days 16 and 19 after the second (or third) AI procedure, using ultrasound scanner with a 5MHz linear array transducer. Females were considered pregnant when an embryonic vesicle or an early conceptus was demonstrated.

### Statistical analysis

Statistical analysis was performed using PASW Statistics software for Windows, version 18.0.0 (Formerly SPSS Statistics; IBM Inc., Chicago, IL, USA). Differences in sperm quality parameters were tested using the Multivariate test within the General Linear Model, where the extender was considered fixed factor. For comparison between the two freezing techniques the paired-sample t-test was used. Comparison between jacks in fresh semen parameters was done using One Way ANOVA. When values were not normally distributed based on Levene Test of Homogeneity of Variances, values were SQRT transformed. The Fisher exact test and the Wilcoxon’s Signed Rank test were used to evaluate differences between groups in pregnancy rate. Differences were considered significant when *P* < 0.05.

## Results

A total of 25 semen collection procedures were performed, five per jack. Despite the fact that the jacks were not collected before the start of the study to deplete old semen from the system, only one sample was deemed unsuitable for cryopreservation (only 10% motility). One sample per jack (last collection) was used for seminal plasma extraction. All other samples were of very good quality (Table 1, S1 Data) and were used in the experiments.

**Table 1:** Fresh donkey semen parameters.

Parameter	Jack				
	1	2	3	4	5
Volume (mL)	39.8 ± 12.3	40.4 ± 7.1	41.2 ± 28.0	26.4 ± 7.5	56.6 ± 6.7
Concentration (× 10^6^)	347 ± 73^ab^	150 ± 47^bc^	340 ± 164^ab^	406 ± 149^a^	31 ± 21^c^
Total motility (%)	72.0 ± 13.5	71.0 ± 4.2	50.0 ± 28.5	63.0 ± 17.9	67.0 ± 13.5
Progressive motility (%)	63.0 ± 17.9	64.0 ± 4.2	45.0 ± 28.5	60.0 ± 17.0	59.0 ± 16.7
Vigour	3.1 ± 0.7	3.2 ± 0.4	3.4 ± 1.1	4.2 ± 0.3	3.5 ± .07
Viability (%)	80.5 ± 9.0	83.0 ± 5.4	84.3 ± 9.4	77.0 ± 12.5	78.8 ± 7.4
Normal morphology (%)	73.3 ± 20.7	88.8 ± 5.9	92.5 ± 7.2	75.8 ± 16.8	74.5 ± 10.7

Values are presented as Mean ± SD. Concentration values with different superscript letter differ significantly at *P* < 0.05. Jacks did not differ in all other parameters evaluated. Values of Total motility and Vigour were not normally distributed (Levene Test of Homogeneity of Variances) so they were SQRT transformed prior to analysis. Values of Total Motility lacked normal distribution even when the sample with 10% motility was removed from analysis.

### Experiment 1: Donkey sperm cryopreservation

Semen was collected successfully from all males on all occasions. One sample was of too low motility to justify freezing. Nineteen samples were processed and frozen. Generally speaking, freezing outcome was fair to good with all samples having post thaw motility of 30% or more in at least one extender/freezing technique.

Comparison between the two freezing techniques revealed that freezing in large-volume HollowTubes^®^ by the directional freezing technique was superior to freezing in straws over liquid nitrogen (Table 2). This was true when the two freezing techniques were compared for each evaluation parameter within each freezing extender and it held true also when data of all freezing extenders were combined (Table 2, S2 Data). The exception to this was the percentage of spermatozoa with normal morphology in which samples frozen in straws were superior to those frozen in tubes when compared for BOTU, INRA, or all samples combined, but not for BC+G.

**Table 2:** Donkey sperm post-thaw evaluation by freezing technique.

Parameter	BC+G		BOTU		INRA		All	
	*DF*	*LNV*	*DF*	*LNV*	*DF*	*LNV*	*DF*	*LNV*
Tot. Motil. %	20.1±10.9*	10.6±8.6*	37.5±10.0*	31.9±10.6*	38.3±6.9*	22.5±10.7*	32.0±12.6*	21.5±13.2*
Prog. Motil. %	12.3±9.7*	6.1±7.0*	32.2±10.2*	26.9±10.7*	35.3±7.4*	20.1±10.8*	26.6±13.6*	17.5±12.9*
Vigour	1.8±0.5	1.6±0.5	2.6±0.6	2.5±0.5	3.2±0.5*	2.9±0.4*	2.5±0.8*	2.3±0.7*
Viability %	81.4±7.8*	72.0±9.4*	77.0±8.8	75.0±7.9	76.6±8.1	70.4±10.2	78.3±8.4*	72.5±9.3*
Morphology %	87.6±7.4	88.4±5.4	84.5±8.9*	89.4±7.0*	86.7±5.2*	90.4±6.2*	86.3±7.3*	89.4±6.1*
3h Motility %	9.5±10.5*	4.2±4.6*	2.6±3.7	1.9±2.3	29.4±10.6*	11.7±8.0*	13.9±14.4*	5.9±6.8*

Values marked with asterisk under the same evaluation parameter and within the same extender differ significantly at *P* < 0.05 (Paired-sample t-test).

df values are: 17 in each extender; 53 when data from all extenders was combined.

Tot. Motil. = Total Motility; Prog. Motil. = Progressive Motility; BC+G = Berliner Cryomedium + Glycerol; BOTU = BotuCrio; INRA = InraFreeze; DF = Directional Freezing; LNV = freezing in the vapour phase above liquid nitrogen.

Comparison (Table 3, S2 Data) showed that there was a statistically significant difference between the three freezing extenders based on post-thaw evaluations [*F* (12, 194) = 27.074, *P* < 0.0005; Wilk’s Λ = 0.140]. Comparison between individual extenders by Post Hoc tests indicated that INRA and BOTU were superior to BC+G in total, progressive and 3 h motility and in vigour (*P* < 0.004 for all). In vigour, INRA was also superior to BOTU (*P* < 0.0005). INRA and BOTU did not differ in total and progressive motility and all three extenders showed similar results when viability and morphology were compared.

**Table 3:** Donkey sperm post-thaw evaluations by extender.

Parameter	BC+G	BOTU	INRA
Tot. Motil. %	15.24 ± 10.79 ^a^	34.72 ± 10.55 ^b^	30.42 ± 11.98 ^b^
Prog. Motil. %	9.14 ± 8.86 ^a^	29.58 ± 10.65 ^b^	27.69 ± 11.91 ^b^
Vigour	1.69 ± 0.52 ^a^	2.56 ± 0.56 ^b^	3.03 ± 0.48 ^c^
Viability %	76.59 ± 9.81	76.00 ± 8.31	73.5 ± 9.59
Morphology %	88.03 ± 6.38	86.97 ± 8.29	88.53 ± 5.92
3h Motility %	6.78 ± 8.33 ^b^	2.25 ± 3.07 ^a^	20.56 ± 12.90 ^c^

Values with different superscript letter in the same row differ significantly at P < 0.05.

Tot. Motil. = Total Motility; Prog. Motil. = Progressive Motility; BC+G = Berliner Cryomedium +Glycerol; BOTU = BotuCrio; INRA = InraFreeze.

As results indicated that BC+G was inferior to the other two extenders, in three ejaculates an attempt was made to reduce the final concentration of glycerol in BC+G from ~4.5% to ~2.7%. Although some improvement was noted, analysis still showed similar picture to the above. With the aim of improving post-thaw motility, we tested the possibility of diluting out the cryoprotectants(s) in the freezing extenders by mixing an aliquot of the thawed sample with the corresponding centrifugation media at a ratio of 1:1. This seemed to improve results for BC+G and BOTU as revealed when motility following 3 h of incubation at 37°C of diluted and undiluted samples was compared (BC+G: t = 2.394, df = 16, *P* = 0.029; BOTU: t = 6.038, df = 16, *P* < 0.0005). Interestingly, for the INRA extender, such dilution caused a drop in motility during the 3 h incubation (t = 3.642, df = 16, *P* = 0.002). It was thus decided that for the second experiment only samples frozen by the directional freezing technique with INRA and BOTU would be used and samples frozen in BOTU would be diluted with BotuSemen after thawing, unless dilution with autologous seminal plasma was done.

### Experiment 2: Artificial insemination

**Fig 2:**
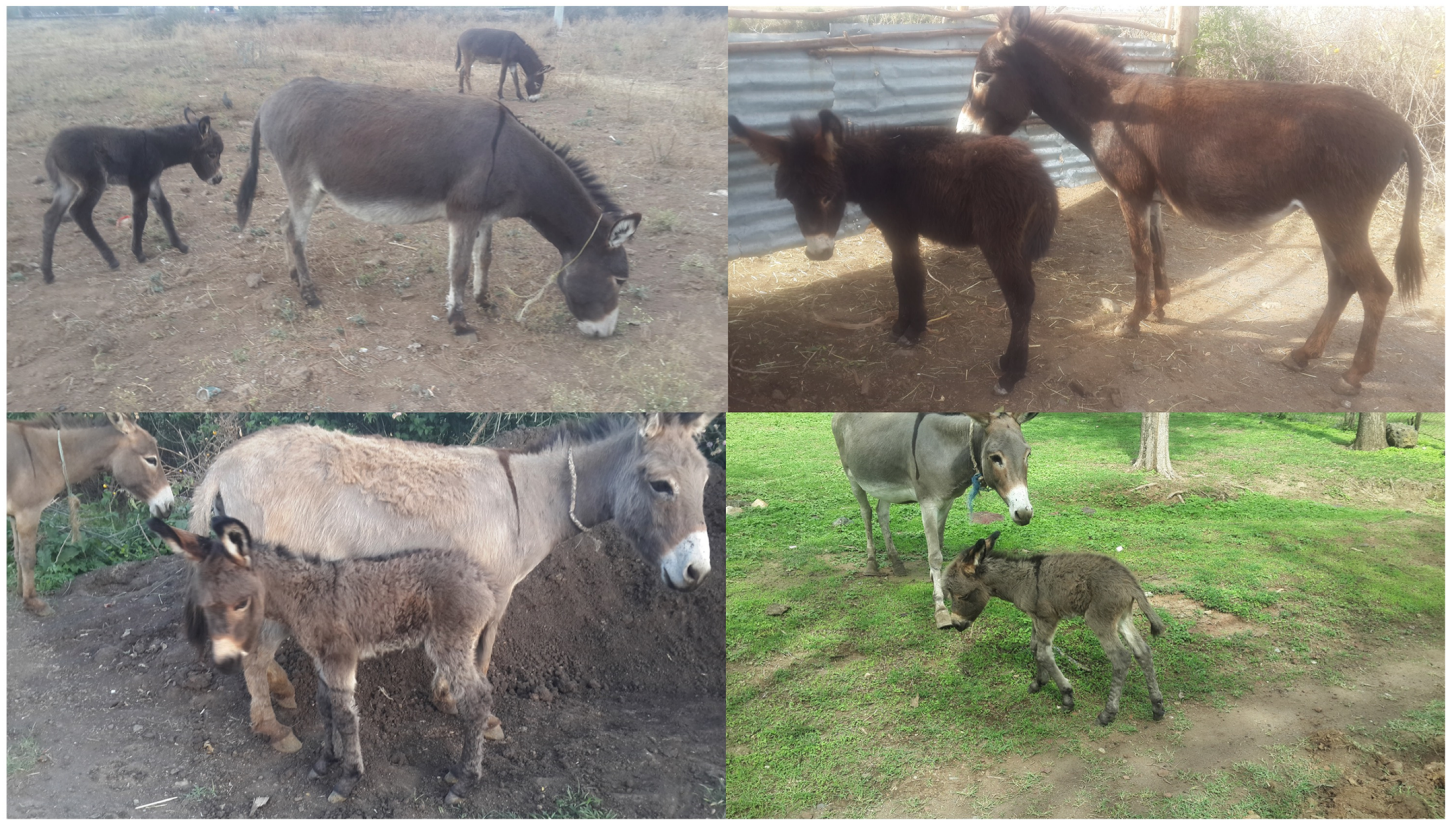
Four of the foals (with their dams) born following artificial insemination performed with frozen-thawed Abyssinian donkey sperm in the present study. No abnormalities were noted in the newborn.

Of the 20 jennies inseminated in this study, nine were confirmed pregnant and in one the ultrasonographic examination suggested lost pregnancy (S3 Data). This last female had matured *corpus luteum* and uterine fluids characteristic for lost pregnancy. We can thus say that all over conception rate was 50%. Of these, five jennies gave birth to five healthy foals (four males and a female; Fig 2). Three females were lost to follow-up (owners moved away or jennies sold), and one has aborted after about two months of pregnancy. Results were analysed in search for possible combinations that resulted in higher pregnancy rate. However, we found no difference between INRA and BOTU, or between insemination doses that were diluted with seminal plasma and those that were not. There was also no effect to the level of sperm motility after thawing (range between 30 and 50%) and no effect to the jennie’s age. When comparing between males, one male stood out as contributing to pregnancy rate lower than the total average (2/7 = 28.6%). Further analysis of the data found that when the pre-ovulatory follicular size was between 33.1 and 37.4 mm, nine of the ten females in this sub-group conceived (90%) whereas only one in the remaining ten jennies conceived. The difference between these two sub-populations was significant (two-tailed Fisher’s exact text, *P* = 0.001). When splitting the jennies based on preovulatory follicular size, we found that the male with the low pregnancy rate happened to have inseminated mainly (5/7) females with follicles larger than 38.5 mm.

## Discussion

“Sancho hastened to his Dapple, and embracing him he said, ‘How hast thou fared, my blessing, Dapple of my eyes, my comrade?’ all the while kissing him and caressing him as if he were a human being. The ass held his peace, and let himself be kissed and caressed by Sancho without answering a single word” [50].

Donkeys have accompanied humans for thousands of years, providing them with a variety of services. Similar to dogs, this human-donkey interaction resulted in a very wide variety of breeds. In recent decades, however, the use of donkeys has drastically dropped and with it came the dwindling of populations, bringing many of these breeds to the verge of extinction or beyond. Protecting this cultural heritage is of paramount importance. Furthermore, in the wild, of the three extant donkey species, two are also under severe threat of extinction. This dire state of donkeys needs to be halted and hopefully reversed. To this end, the present study was devised, targeting a long-standing hurdle faced by assisted reproduction in donkeys. Whereas freezing donkey sperm seem to be working well, using frozen-thawed donkey sperm to inseminate jennies result in consistently low conception rates, ranging between zero and about 36% [12,13,19,51] with just one report showing higher conception rate of about 62% [26]. Despite a number of studies on the topic over the last 20 years or so, it remains unknown why frozen donkey sperm does not succeed in fertilizing the jennies. It has been hypothesised that the culprit is with the glycerol in the freezing extender. One line of thought suggested that glycerol is toxic to donkeys’ sperm [12] while another suggested that it is the jennie’s uterine environment that is sensitive to the compound [13]. The latter study even showed that pregnancy rate was higher in mares inseminated with frozen-thawed donkey sperm than it was in jennies, further supporting the theory that the jennies’ sensitivity to glycerol stand behind the low pregnancy rates observed in donkeys. If this explanation was correct, however, one would have expected proper conception rates when other cryoprotectants were used. Yet, this was not really the case [19]. Freezing with glycerol but removing it prior to insemination did improve things in a study on the Poitou donkey but pregnancy rate was zero in its presence making comparison questionable [12]. In our study we found no effect to the dilution out of the glycerol with autologous seminal plasma (INRA) or using glycerol-free extender (BOTU). All these suggest that glycerol might not be the factor affecting conception rate in jennies inseminated with frozen-thawed donkey sperm. An alternative explanation for the better success rate achieved thus far when frozen-thawed donkey sperm was used to inseminate mares is because the knowledge of the mare reproductive biology, cycle and follicular growth dynamics, and when is the ideal insemination window are better known compared to those of the jenny and so inseminations in those studies possibly took place at a more suitable time. It was previously reported in other donkey breeds (Nordestina or other undetermined breed) that seasonality had no effect on the jack’s sexual behaviour [52] or ejaculate characteristics [53]. It would help to find out if the same applies to the Abyssinian donkey jacks in Ethiopia, as this information will indicate if there is optimal time of the year for semen collection and preservation. The same studies, while noting difference between jacks, found no difference between the two weekly semen collections. In our study we noted variations between jacks only in ejaculate concentration. In some jacks, between-ejaculates differences were apparent but we noted no relation between collection sequence and ejaculate parameters.

In some species, e.g. llama [54] or pig [55], addition of seminal plasma to the thawed sperm before insemination seems to improve fertilisation rate. In donkeys, the addition of seminal plasma to post-thaw sperm showed no improvement in *in vitro* sperm characteristics [27] and, as shown in our study, had no effect on pregnancy rate, when it was added to the thawed sample before insemination. This is yet another demonstration of the fact that species are different from each other and thus require species-specific customisation of protocols.

In an attempt to resolve the issue at hand, we tried to use different freezing extenders, containing different cryoprotectants, and to use the directional freezing—a freezing technique never tested in donkeys. Looking at the results, however, nothing of all these, or the males themselves, seems to have any effect on conception rate. With 50%, conception rate was at the higher end of what thus far has been achieved in donkeys and on par with what is customary in horses [56,57] or the highly-managed dairy and beef cattle industry (e.g. [58]). Considering the fact that most of the jennies in this study were working animals with less-than-ideal nutrition, 50% pregnancy rate is a great outcome. We did, however, hope for more. It was only when we looked at the association between conception rate and pre-ovulatory follicular size that we got a clue as to how pregnancy rates in donkeys inseminated with frozen-thawed donkey sperm may be improved. The association between pre-ovulatory follicular size and conception rate has been reported before in other species [59,60] and it was suggested that when ovulation is induced, some immature follicles are ovulated, leading to lower pregnancy rates [59]. Still, the general association seems to be one that binds between follicular size, follicular maturation, and pregnancy rate. In other words, the larger the follicle, the higher pregnancy rate in induced ovulation cycles [60,61]. In our study ovulation was not induced. All jennies ovulated naturally. It could certainly be a matter of numbers as 20 jennies are not a large enough sample to provide a full picture of the population, but it could also be a species-specific character. Follicles smaller than 33.1 mm may have ovulated before they had properly matured and were thus less likely to result in pregnancy. Why follicles larger than the higher end of the range (37.4 mm), with the exception of the largest follicle observed in this study (41.3 mm), did not result in pregnancy is not known. Based on our previous [62,63] and the present study’s results, the Ethiopian long (June to September) and short (March to May) rainy seasons seem to be the right mating seasons for these jennies when average pre-ovulatory follicles were measured at 33.2±1.2 mm and 37.8±2.1 mm, respectively. During the dry season (October to February), when conditions are tough, the pre-ovulatory follicles are smaller (31.0±1.8) thus appear to be rendering the jennies with lower probability of getting pregnant.

It has been suggested that when ovulation is induced, supporting hormones, such as estradiol, do not reach their optimal levels [59]. This, however, was not the case in our study, as we relied on natural ovulation. Inducing the cycle may have its affect on the hormonal status of the females and thus on follicular development and maturation. A future study, evaluating the hormonal status of the jennies before, at, and after ovulation and insemination, may provide the answer. To make sure that all jennies were inseminated in proximity of ovulation, two jennies in our study were inseminated three times—twice, ~24h apart, before ovulation and once after ovulation as opposed to once before and once after in all other jennies. If this had an effect on the success rate we do not know but one should keep this in mind when interpreting the results of our study.

This study provides a further confirmation to the superiority of the directional freezing technique over freezing in straws in nitrogen vapour. We and others have shown this to be the case in a wide variety of species including domestic species such as bovine bull [32] and stallion [34], and non-domestic species such as rhinoceros [44], dolphin [42], killer whale [64], and most recently also in the onager [29]. The most plausible explanation for this outcome is the different way ice crystals grow within the container in these two techniques—stochastic with possible recrystallisation in straws over liquid nitrogen vs. highly organised and controlled ice crystal morphology and efficient heat dissipation in tubes frozen by the directional freezing technique [65]. The result is a much better protection of the delicate cells inside the solution when directional freezing is used.

In conclusion, in this study we have demonstrated that when freezing donkey sperm the directional freezing was superior to freezing in straws over liquid nitrogen, and that INRA and BOTU were the freezing extenders of choice. Pre-ovulatory follicular size was found to be a reliable predictor for successful AI. The protocols developed through this project will contribute to a long-standing deficiency in wildlife management, namely the ability to artificially inseminate endangered donkey species and breeds, including the Senar donkey in Amhara Regional State in Ethiopia. With this knowledge in hand, the captive populations and isolated wild populations will gain access to unrepresented genetic material, thus improving their resilience and sustainability in face of inbreeding and its negative consequences. It would, however, be beneficial to conduct a large-scale study to reconfirm the preliminary findings of this study and their applicability to the donkey population.

## Supporting information

S1 Data Evaluation data of fresh Abyssinian donkey semen. (XLS)

S2 Data Evaluation data of frozen-thawed Abyssinian donkey spermatozoa. (XLS)

S3 Data Data on pre-ovulatory follicular size and outcome of artificial insemination with frozen-thawed sperm in Abyssinian donkeys. (XLS)

## References

[1] FAO (2007) The State of the World's Animal Genetic Resources for Food and Agriculture. Rome: Food and Agriculture Organization of the United Nations 524 p.

[2] Moehlman PD, Shah N, Feh C (2008) Equus hemionus. The IUCN Red List of Threatened Species. Version 2014.3. <www.iucnredlist.org>. Accessed on: Downloaded on 13 January 2015.

[3] Moehlman PD, Yohannes H, Teclai R, Kebede F (2008) Equus africanus. The IUCN Red List of Threatened Species. Version 2014.3. <www.iucnredlist.org>. Accessed on: Downloaded on 13 January 2015.

[4] Saragusty J, Diecke S, Drukker M, Durrant B, Friedrich Ben-Nun I, Galli C, et al (2016) Rewinding the process of mammalian extinction. Zoo Biol 35: 280–292. 10.1002/zoo.21284 27142508

[5] Andrabi SMH, Maxwell WMC (2007) A review on reproductive biotechnologies for conservation of endangered mammalian species. Anim Reprod Sci 99: 223–243. 10.1016/j.anireprosci.2006.07.002 16919407

[6] Pukazhenthi BS, Wildt DE (2004) Which reproductive technologies are most relevant to studying, managing and conserving wildlife? Reprod Fertil Dev 16: 33–46. 14972101 10.10371/RD03076

[7] Hildebrandt TB, Hermes R, Saragusty J, Potier R, Schwammer HM, Balfanz F, et al (2012) Enriching the captive elephant population genetic pool through artificial insemination with frozen-thawed semen collected in the wild. Theriogenology 78: 1398–1404. 10.1016/j.theriogenology.2012.06.014 22898009

[8] Hildebrandt TB, Roellig K, Goeritz F, Fassbender M, Krieg R, Blottner S, et al (2009) Artificial insemination of captive European brown hares (*Lepus europaeus* PALLAS, 1778) with fresh and cryopreserved semen derived from free-ranging males. Theriogenology 72: 1065–1072. 10.1016/j.theriogenology.2009.06.026 19740536

[9] Barker CAV, Gandier JCC (1957) Pregnancy in a mare resulted from frozen epididymal spermatozoa. Can J Comp Med Vet Sci 21: 47–51. 17648942 PMC1614368

[10] Trimeche A, Anton M, Renard P, Gandemer G, Tainturier D (1997) Quail egg yolk: a novel cryoprotectant for the freeze preservation of Poitou jackass sperm. Cryobiology 34: 385–393. 10.1006/cryo.1997.2009 9200823

[11] Trimeche A, Renard P, Le Lannou D, Barriere P, Tainturier D (1996) Improvement of motility of post-thaw Poitou jackass sperm using glutamine. Theriogenology 45: 1015–1027. 16727860 10.1016/0093-691x(96)00029-5

[12] Trimeche A, Renard P, Tainturier D (1998) A procedure for Poitou jackass sperm cryopreservation. Theriogenology 50: 793–806. 10734453 10.1016/s0093-691x(98)00184-8

[13] Vidament M, Vincent P, Martin FX, Magistrini M, Blesbois E (2009) Differences in ability of jennies and mares to conceive with cooled and frozen semen containing glycerol or not. Anim Reprod Sci 112: 22–35. 10.1016/j.anireprosci.2008.03.016 18502059

[14] Vidament M, Vincent P, Yvon JM, Bruneau B, Martin FX (2005) Glycerol in semen extender is a limiting factor in the fertility in asine and equine species. Anim Reprod Sci 89: 302–305 (abstract). 16265750

[15] Álvarez AL, Serres C, Torres P, Crespo F, Mateos E, Gómez-Cuétara C (2006) Effect of cholesterol-loaded cyclodextrin on the cryopreservation of donkey spermatozoa. Anim Reprod Sci 94: 89–91 (abstract).

[16] Cortés-Gutiérrez EI, Crespo F, Gosálvez A, Dávila-Rodríguez MI, López-Fernández C, Gósalvez J (2008) DNA fragmentation in frozen sperm of Equus asinus: Zamorano-Leonés, a breed at risk of extinction. Theriogenology 69: 1022–1032. 10.1016/j.theriogenology.2008.02.002 18367243

[17] Canisso IF, Carvalho GR, Morel MCGD, Guimarães JD, McDonnell SM (2010) Sexual behavior and ejaculate characteristics in Pêga donkeys (*Equus asinus*) mounting estrous horse mares (*Equus caballus*). Theriogenology 73: 56–63. 10.1016/j.theriogenology.2009.07.026 19775738

[18] Canisso IF, Carvalho GR, Morel MD, Ker PG, Rodrigues AL, Silva EC, et al (2011) Seminal parameters and field fertility of cryopreserved donkey jack semen after insemination of horse mares. Equine Vet J 43: 179–183. 10.1111/j.2042-3306.2010.00130.x 21592212

[19] Oliveira JV, Alvarenga MA, Melo CM, Macedo LM, Dell'aqua JA Jr., Papa FO (2006) Effect of cryoprotectant on donkey semen freezability and fertility. Anim Reprod Sci 94: 82–84 (abstract).

[20] Contri A, De Amicis I, Veronesi MC, Faustini M, Robbe D, Carluccio A (2010) Efficiency of different extenders on cooled semen collected during long and short day length seasons in Martina Franca donkey. Anim Reprod Sci 120: 136–141. 10.1016/j.anireprosci.2010.02.018 20346604

[21] Contri A, Gloria A, Robbe D, Sfirro MP, Carluccio A (2012) Effect of sperm concentration on characteristics of frozen-thawed semen in donkeys. Anim Reprod Sci 136: 74–80. 10.1016/j.anireprosci.2012.10.022 23182476

[22] Miró J, Lobo V, Quintero-Moreno A, Medrano A, Peña A, Rigau T (2005) Sperm motility patterns and metabolism in Catalonian donkey semen. Theriogenology 63: 1706–1716. 10.1016/j.theriogenology.2004.07.022 15763113

[23] Ortiz I, Dorado J, Acha D, Gálvez MJ, Urbano M, Hidalgo M (2013) Colloid single-layer centrifugation improves post-thaw donkey (*Equus asinus*) sperm quality and is related to ejaculate freezability. Reprod Fertil Dev 27: 332–340. 10.1071/RD13246 25482321

[24] Qeusada F, Dorado J, Acha D, Ortiz I, Urbano M, Ramirez L, et al (2012) Freezing of donkey semen after 24 hours of cool storage: Preliminary results. Reprod Fertil Dev 25: 154 (abstract).

[25] Acha D, Hidalgo M, Ortiz I, Gálvez MJ, Carrasco JJ, Gómez-Arrones V, et al (2015) Freezability of Andalusian donkey (*Equus asinus*) spermatozoa: effect of extenders and permeating cryoprotectants. Reprod Fertil Dev 28: 1990–1998. 10.1071/RD14449 26129907

[26] Rota A, Panzani D, Sabatini C, Camillo F (2012) Donkey jack (*Equus asinus*) semen cryopreservation: studies of seminal parameters, post breeding inflammatory response, and fertility in donkey jennies. Theriogenology 78: 1846–1854. 10.1016/j.theriogenology.2012.07.015 22979965

[27] Sabatini C, Mari G, Mislei B, Love CC, Panzani D, Camillo F, et al (2014) Effect of post-thaw addition of seminal plasma on motility, viability and chromatin integrity of cryopreserved donkey jack (*Equus asinus*) spermatozoa. Reprod Domest Anim 49: 989–994. 10.1111/rda.12419 25256158

[28] Jepsen RJ, Evans LE, Youngs CR (2010) Use of direct thaw insemination to establish pregnancies with frozen-thawed semen from a standard jack. J Equine Vet Sci 30: 651–656.

[29] Prieto Pablos MT, Saragusty J, Santiago-Moreno J, Stagegaard J, Göritz F, Hildebrandt TB, et al (2015) Cryopreservation of onager (*Equus hemionus onager*) epididymal spermatozoa. J Zoo Wildl Med 46: 517–525. 10.1638/2014-0243.1 26352955

[30] Schook MW, Wildt DE, Weiss RB, Wolfe BA, Archibald KE, Pukazhenthi BS (2013) Fundamental studies on the reproductive biology of the endangered Persian onager (*Equus hemionus onager*) result in first wild equid offspring from artificial insemination. Biol Reprod 89: 1–13. 10.1095/biolreprod.113.110122 23863403

[31] Alghamdi AS, Madill S, Foster DN (2005) Seminal plasma improves fertility of frozen equine semen. Anim Reprod Sci 89: 242–245 (abstract). 16265727

[32] Hayakawa H, Yamazaki T, Oshi M, Hoshino M, Dochi O, Koyama H (2007) Cryopreservation of conventional and sex-sorted bull sperm using a directional freezing method. Reprod Fertil Dev 19: 176–177 (abstract).

[33] Saragusty J, Gacitua H, Zeron Y, Rozenboim I, Arav A (2009) Double freezing of bovine semen. Anim Reprod Sci 115: 10–17. 10.1016/j.anireprosci.2008.11.005 19108961

[34] Saragusty J, Gacitua H, Pettit MT, Arav A (2007) Directional freezing of equine semen in large volumes. Reprod Domest Anim 42: 610–615. 10.1111/j.1439-0531.2006.00831.x 17976068

[35] Gacitua H, Arav A (2005) The effect of sperm number per insemination of fresh and cryopreserved Saanen buck semen. Reprod Domest Anim 40: 383 (abstract).

[36] Saragusty J, Hildebrandt TB, Behr B, Knieriem A, Kruse J, Hermes R (2009) Successful cryopreservation of Asian elephant (*Elephas maximus*) spermatozoa. Anim Reprod Sci 115: 255–266. 10.1016/j.anireprosci.2008.11.010 19111407

[37] Hermes R, Saragusty J, Göritz F, Bartels P, Potier R, Baker B, et al (2013) Freezing African elephant semen as a new population management tool. PLoS ONE 8: e57616 10.1371/journal.pone.0057616 23483917 PMC3590205

[38] Saragusty J, Gacitua H, King R, Arav A (2006) Post-mortem semen cryopreservation and characterization in two different endangered gazelle species (*Gazella gazella and Gazella dorcas*) and one subspecies (*Gazella gazelle acaiae*). Theriogenology 66: 775–784. 10.1016/j.theriogenology.2006.01.055 16530260

[39] Saragusty J, Walzer C, Petit T, Stalder G, Horowitz I, Hermes R (2010) Cooling and freezing of epididymal sperm in the common hippopotamus (*Hippopotamus amphibius*). Theriogenology 74: 1256–1263. 10.1016/j.theriogenology.2010.05.031 20615541

[40] Hermes R, Göritz F, Saragusty J, Sos E, Molnar V, Reid CE, et al (2009) First successful artificial insemination with frozen-thawed semen in rhinoceros. Theriogenology 71: 393–399. 10.1016/j.theriogenology.2008.10.008 19007979

[41] Robeck TR, Steinman KJ, Montano GA, Katsumata E, Osborn S, Dalton L, et al (2010) Deep intra-uterine artificial inseminations using cryopreserved spermatozoa in beluga (*Delphinapterus leucas*). Theriogenology 74: 989–1001. 10.1016/j.theriogenology.2010.04.028 20570326

[42] O'Brien JK, Robeck TR (2006) Development of sperm sexing and associated assisted reproductive technology for sex preselection of captive bottlenose dolphins (*Tursiops truncatus*). Reprod Fertil Dev 18: 319–329. 16554007 10.1071/rd05108

[43] Arav A, Saragusty J (2013) Directional freezing of spermatozoa and embryos. Reprod Fertil Dev 26: 83–90. 10.1071/RD13295 24305180

[44] Reid CE, Hermes R, Blottner S, Goeritz F, Wibbelt G, Walzer C, et al (2009) Split-sample comparison of directional and liquid nitrogen vapour freezing method on post-thaw semen quality in white rhinoceroses (*Ceratotherium simum simum* and *Ceratotherium simum cottoni*). Theriogenology 71: 275–291. 10.1016/j.theriogenology.2008.07.009 18775559

[45] Crane M (1997) Medical In: Svendsen ED, editor. The professional Handbook of the Donkey. 3rd ed London, UK: Whittet Books pp. 19–35.

[46] Pearson RA, Ouassat M (2000) A Guide to Live Weight Estimation and Body Condition Scoring of Donkeys. Edinburgh: Centre for Tropical Veterinary Medicine, University of Edinburgh 21 p.

[47] Blottner S (1998) Semen preservation for reproduction management in rare and endangered species. Adv Ethol 33: 9–13.

[48] Saragusty J, Gacitua H, Rozenboim I, Arav A (2009) Protective effects of iodixanol during bovine sperm cryopreservation. Theriogenology 71: 1425–1432. 10.1016/j.theriogenology.2009.01.019 19299004

[49] Saragusty J (2015) Directional freezing for large volume cryopreservation In: Wolkers WF, Oldenhof H, editors. Methods in Cryopreservation and Freeze-Drying. New York: Springer Verlarg pp. 381–397. 10.1007/978-1-4939-2193-5_19 25428019

[50] de Cervantes M (1605) Don Quixote. Adelaide, Australia: The University of Adelide.

[51] Rota A, Magelli C, Panzani D, Camillo F (2008) Effect of extender, centrifugation and removal of seminal plasma on cooled-preserved Amiata donkey spermatozoa. Theriogenology 69: 176–185. 10.1016/j.theriogenology.2007.09.003 17945340

[52] Gastal MO, Henry M, Beker AR, Gastal EL, Goncalves A (1996) Sexual behavior of donkey jacks: Influence of ejaculatory frequency and season. Theriogenology 46: 593–603. 10.1016/0093-691X(96)00211-7 16727925

[53] Gastal MO, Henry M, Beker AR, Gastal EL (1997) Effect of ejaculation frequency and season on donkey jack semen. Theriogenology 47: 627–638. 16728015 10.1016/s0093-691x(97)00021-6

[54] Tanco VM, Ratto MH, Lazzarotto M, Adams GP (2011) Dose-response of female llamas to ovulation-inducing factor from seminal plasma. Biol Reprod 85: 452–456. 10.1095/biolreprod.111.091876 21593475

[55] Okazaki T, Akiyoshi T, Kan M, Mori M, Teshima H, Shimada M (2012) Artificial insemination with seminal plasma improves the reproductive performance of frozen-thawed boar epididymal spermatozoa. J Androl 33: 990–998. 10.2164/jandrol.111.015115 22282435

[56] Vidament M (2005) French field results (1985–2005) on factors affecting fertility of frozen stallion semen. Anim Reprod Sci 89: 115–136. 10.1016/j.anireprosci.2005.07.003 16112529

[57] Loomis PR (2001) The equine frozen semen industry. Anim Reprod Sci 68: 191–200. 11744264 10.1016/s0378-4320(01)00156-7

[58] Sales JNS, Neves KAL, Souza AH, Crepaldi GA, Sala RV, Fosado M, et al (2011) Timing of insemination and fertility in dairy and beef cattle receiving timed artificial insemination using sex-sorted sperm. Theriogenology 76: 427–435. 10.1016/j.theriogenology.2011.02.019 21497392

[59] Perry GA, Smith MF, Lucy MC, Green JA, Parks TE, MacNeil MD, et al (2005) Relationship between follicle size at insemination and pregnancy success. Proc Nat Acad Sci USA 102: 5268–5273. 10.1073/pnas.0501700102 15795381 PMC556005

[60] Sá Filho MF, Crespilho AM, Santos JEP, Perry GA, Baruselli PS (2010) Ovarian follicle diameter at timed insemination and estrous response influence likelihood of ovulation and pregnancy after estrous synchronization with progesterone or progestin-based protocols in suckled *Bos indicus* cows. Anim Reprod Sci 120: 23–30. 10.1016/j.anireprosci.2010.03.007 20395079

[61] Rahman MS, Shohag AS, Kamal MM, Bari FY, Shamsuddin M (2012) Preovulatory follicular and subsequent luteal size influence pregnancy success in water buffaloes. J Reprod Dev 58: 219–222. 22156378 10.1262/jrd.11-111t

[62] Lemma A, Bekana M, Schwartz HJ, Hildebrandt T (2006) Ultrasonographic study of ovarian activities in the tropical jenny during the seasons of high and low sexual activity. J Equine Vet Sci 26: 18–22.

[63] Lemma A, Bekana M, Schwartz HJ, Hildebrandt T (2006) The effect of body condition on ovarian activity of free ranging tropical jennies (*Equus asinus*). Journal of Veterinary Medicine Series A-Physiology Pathology Clinical Medicine 53: 1–4. 10.1111/j.1439-0442.2006.00777.x 16411899

[64] Robeck TR, Gearhart SA, Steinman KJ, Katsumata E, Loureiro JD, O'Brien JK (2011) *In vitro* sperm characterization and development of a sperm cryopreservation method using directional solidification in the killer whale (*Orcinus orca*). Theriogenology 76: 267–279. 10.1016/j.theriogenology.2011.02.003 21496896

[65] Saragusty J, Gacitua H, Rozenboim I, Arav A (2009) Do physical forces contribute to cryodamage? Biotechnol Bioeng 104: 719–728. 10.1002/bit.22435 19593758

